# Plant Ribosome-Inactivating Proteins: Progesses, Challenges and Biotechnological Applications (and a Few Digressions)

**DOI:** 10.3390/toxins9100314

**Published:** 2017-10-12

**Authors:** Maria Serena Fabbrini, Miku Katayama, Ikuhiko Nakase, Riccardo Vago

**Affiliations:** 1 MIUR, Italian Ministry of Instruction, University and Research, 20090 Monza, Italy; msfabbrini@gmail.com; 2 NanoSquare Research Institution, Research Center for the 21st Century, Organization for Research Promotion, Osaka Prefecture University, 1-2, Gakuen-cho, Naka-ku, Osaka 599-8570, Japan; sxc04031@edu.osakafu-u.ac.jp (M.K.); i-nakase@21c.osakafu-u.ac.jp (I.N.); 3 Graduate School of Science, Osaka Prefecture University, 1-1, Gakuen-cho, Naka-ku, Osaka 599-8531, Japan; 4 Urological Research Institute, Division of Experimental Oncology, IRCCS San Raffaele Hospital, 20132 Milan, Italy; 5 University Vita-Salute San Raffaele, 23132 Milan, Italy

**Keywords:** plant ribosome inactivating proteins, ER-stress, saporin, targeted drug delivery, nanovectors

## Abstract

Plant ribosome-inactivating protein (RIP) toxins are EC3.2.2.22 N-glycosidases, found among most plant species encoded as small gene families, distributed in several tissues being endowed with defensive functions against fungal or viral infections. The two main plant RIP classes include type I (monomeric) and type II (dimeric) as the prototype ricin holotoxin from *Ricinus communis* that is composed of a catalytic active A chain linked via a disulphide bridge to a B-lectin domain that mediates efficient endocytosis in eukaryotic cells. Plant RIPs can recognize a universally conserved stem-loop, known as the α-sarcin/ ricin loop or SRL structure in 23S/25S/28S rRNA. By depurinating a single adenine (A4324 in 28S rat rRNA), they can irreversibly arrest protein translation and trigger cell death in the intoxicated mammalian cell. Besides their useful application as potential weapons against infected/tumor cells, ricin was also used in bio-terroristic attacks and, as such, constitutes a major concern. In this review, we aim to summarize past studies and more recent progresses made studying plant RIPs and discuss successful approaches that might help overcoming some of the bottlenecks encountered during the development of their biomedical applications.

## 1. Prologue

The first time I heard the term ribosome-inactivating protein “RIP” was in 1987 when we were attending “GENE87” at the Milan University and one of the invited speakers was Prof. Fiorenzo Stirpe from the University ”Alma Mater” of Bologna [1]. The speech was fascinating to all of us coming to attend the symposium from a Plant Biology institute. I had just started my own experimental thesis and it was even more intriguing that Prof. Stirpe was coming from a Medical School and not from a Botanical Institute. The bright idea of using plant-derived toxins to eliminate transformed cells was pioneered at that time. The two seminal papers by Endo and Tsurugi on the mechanism of action of ricin and type I RIPs acting on eukaryotic ribosomes were published this very same year [2,3]. Curiously, some researchers from an Italian pharmaceutical company came to our lab to get some advice on how to achieve the cloning of a RIP cDNA from *Saponaria officinalis* tissues. The dry seeds they were trying to use for preparing the cDNA library stored plenty of saponins that during the mashing procedures were producing huge amounts of bubbles (L. Benatti, personal communication). This is the main reason why the first saporin cDNA was then cloned starting from fresh leaves [4], allowing me just by chance to meet the person with whom we still are sharing our lives. To end these digressions, we must certainly acknowledge the great amount of experimental work done by the group of Mike Lord and Lynne Roberts in Warwick while studying ricin, the prototype type II RIP, one of the most potent poisons known at that time, which was strikingly used to assassinate in a “rocambolesque” way a dissident in London during the heavy years of the cold war. Plant ribosome-inactivating proteins may be viewed as very special tools from the Plant Kingdom that allowed us to shed light on certain peculiar intracellular pathways, such as the retrograde transport along the secretory route or more recent findings about some RIP signal peptide(s) acting as stress-sensors. Still intracellular pathways of delivery need to be elucidated in detail to allow in the future more efficient uses in targeted anticancer therapy.

## 2. Biochemical and Structural Considerations

Several plant species belonging to 17 different families, among them Cucurbitaceae, Euphorbiaceae, and Poaceae, and families belonging to the superorder Caryophyllales, produce plant Ribosome-Inactivating Proteins (RIPs), although many others do not, including the plant type model *Arabidopsis thaliana* [5]. They are found in most plant species as gene families, reflecting their differential distribution in plant tissues (roots, leaves and seeds) and may share among major functions the protection against viral or fungal infections and possibly be relevant for the physiologic responses during plant senescence or following stress inducers [6,7]. RIPs belong to the N-glycosidase family of toxins (EC3.2.2.22) able to specifically and irreversibly inactivate the large ribosomal subunits depurinating a specific adenine base (A4324 in the rat 28S ribosomal rRNA) located in a universally conserved GAGA-tetraloop, also known as the α-sarcin/ricin loop, present in 23S/26S/28S rRNA. Plant RIPs can be divided into three main classes: type I like saporin from *Saponaria officinalis* are composed of a single polypeptide chain of approximately 30 KDa, type II as ricin from *Ricinus communis* [8] are heterodimers consisting of an A chain, functionally equivalent to the type I polypeptide linked via a disulphide bridge to a B subunit endowed with lectin-binding properties [9]. For a long time, all type 2 RIPs were considered to be highly potent toxins, but, so far, there are also known type II RIPs, which are not or only less toxic in vivo, and therefore they are denominated as non-toxic type II RIPs [10,11]. Finally, type III RIPs are polypeptides, which are synthesized as inactive precursors (ProRIPs) that will require proteolytic processing events to form an active RIP [12].

**Figure 1:**
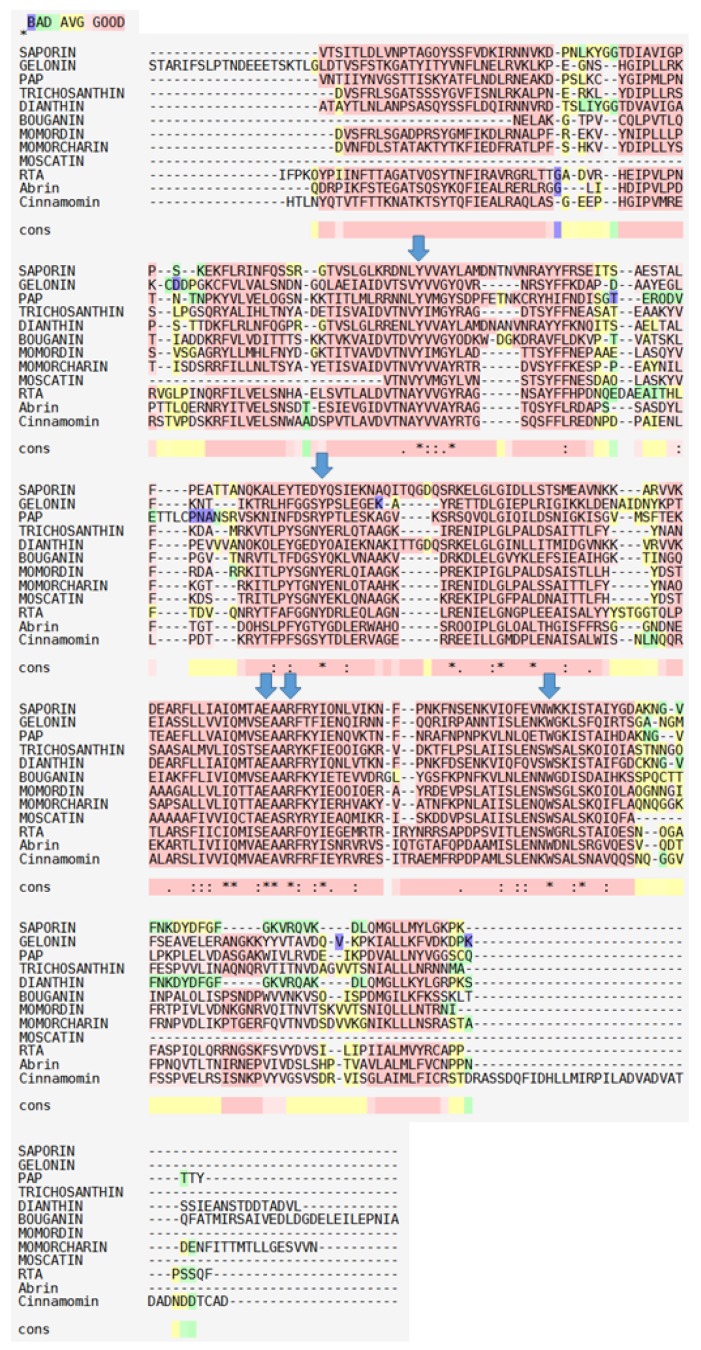
Amino acid sequence alignment of different type I RIPs compared to ricin (RTA), abrin and cinnamomin catalytic A chains by T-Coffee. Color shades indicate levels of amino acid homology between the aligned sequences. Conserved amino acids are identified with an asterisk and residues crucial for the catalytic activity are arrowed: Tyr72, Tyr120, Glu176, Arg179 and Trp208 in the sequence of saporin.

**Figure 2:**
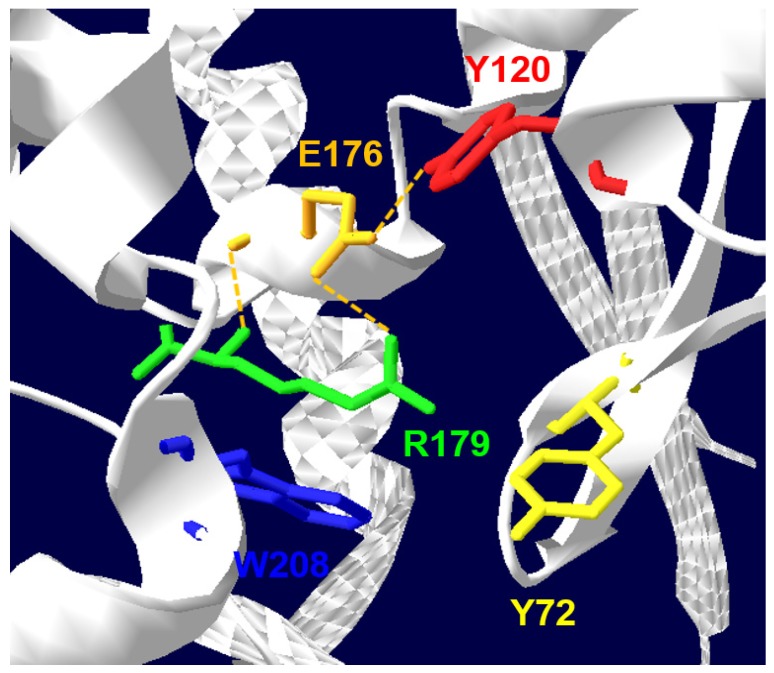
Three-dimensional reconstruction of catalytic cleft of saporin obtained by Swiss PDB Viewer (v4.0.4, SIB—Swiss Institute of Bioinformatics, Lausanne, Switzerland). Conserved residues crucial for the RIP signature are colored: Tyr72 (yellow), Tyr120 (red), Glu176 (orange), Arg179 (green) and Trp208 (blue). Hydrogen bonds among key residues are shown in orange.

Residues that are highly conserved among RIPs (shown in Figure 1 with an asterisk), besides the main residues at the catalytic cleft (arrowed in Figure 1), are those belonging to the “N-glycosidase signature”, which include Tyr80, Tyr123, and the key active site residues Glu177, Arg180, and Trp211 in RTA (Figure 2) and a few others surrounding this active site. The protein sequence identities between ricin A chain (RTA) and type I RIPs (Figure 1) are generally low and found to be respectively: saporin 22%, Gelonin 30%, pokeweed antiviral protein (PAP) 29%, thricosanthin 35%, dianthin 19%, bouganin 29%, and momordin / momorcharin, 33%.

**Figure 3:**
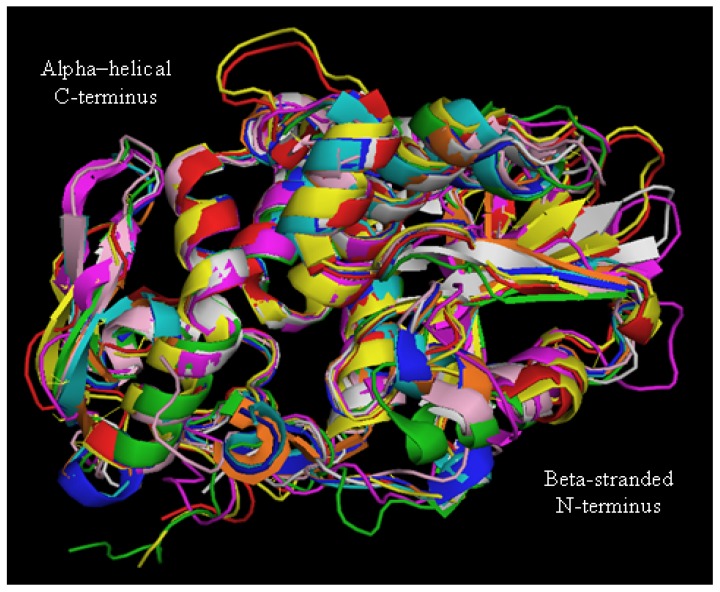
Three-dimensional structure of different type I RIPs and ricin A chain (RTA). Superimposition of secondary structure elements of Saporin (*red*, PDB code 1QI7), Gelonin (*pink*, 3KU0), PAP (*magenta*, 1GIK), Trichosanthin (*cyan*, 1QD2), Dianthin (*yellow*, 1RL0), Bouganin (*grey*, 3CTK), Momordin (*orange*, 1 MOM), Momorcharin (*blue*, 1AHA), RTA (*green*, 1J1 M), modified from [31].

Despite the differences in amino acid sequences, their overall three-dimensional fold is well conserved as estimated by the superimposition of the 3D structures of several type I RIPs with the one of RTA (Figure 3), which clearly demonstrates that RTA and type I RIPs all share a common “RIP fold” characterized by the presence of two major domains: an N-terminal domain, which is mainly beta-stranded, and a C-terminal domain that is predominantly alpha–helical.

Insertions and deletions, as compared to PAP, momordin from *Momordica charantia L*. and RTA were found to lay mainly in random coil regions. Glu177 and Arg180 in RTA (as Glu176 and Arg179 in saporin) are directly involved in the mechanism of catalysis. However, while a RTA Glu177 mutant was 20-fold less active than wild-type A chain in inhibiting translation in a reticulocyte lysate, the Arg179 saporin mutant was found 200-fold less active [13]. Double mutants at the catalytic site have been investigated for several type I RIPs, since for heterologous expression studies less active site mutants were needed to allow elucidating their biosynthesis. A loss of in vitro and in vivo saporin cytotoxicity can be achieved when Glu176 and Arg179 are both mutated to lysine and glutamine residues, respectively. This double saporin mutant (termed KQ) is, indeed, devoid of all the detrimental effects associated with RIP expression in several hosts [14,15,16,17]. Interestingly, mutation of Trp208 in saporin did not impair its in vitro enzymatic activity nor cytotoxicity [18], but this same residue has been demonstrated to be crucial for PAP structural integrity [19]. A negative electrostatic potential, arising from both the negatively charged phosphodiester backbone and conserved solvent-exposed acidic patches on the ribosomal proteins, covers much of the ribosomal surface [20]. The net positive charge of saporin and its high content in basic residues (around 10% lysine residues) are likely to be critical for the recognition of the ribosomal surface. In RTA, a set of arginine residues around the active site are involved in electrostatic interactions with the phosphodiester backbone of the α-sarcin/ricin loop [21,22]. Both RTA- and saporin-catalyzed rRNA modification shows a net dependence on salt and ion concentrations, indicating that these toxins can exploit multiple electrostatic interactions with their target ribosomes [23]. Extra enzymatic activities have been putatively ascribed to RIPs, but apart the Polynucleotide: adenosineglycosidase (PAG) activity documented as a DNA multiple depurination, DNAse-like and RNAse-like activities seem to be due to cross-contaminations of the protein preparation [15,24,25,26,27]. Similar observations were also recently made [28], when comparing saporin-6 to the leaf isoform L1/L3 (which behaves differently from all other isoforms studied to date), they showed that saporin-6 enzymatic activity released two adenines from ribosomes, the major fraction being the one deriving from the N-glycosidase activity while L1/L3 was able to “multidepurinate” ribosomes. Characterization of the kinetic parameters indicated that poly(A) RNA depurination proceeds with a Km of 639 ± 32 µM and a kcat of 61 ± 1 min^−1^ at pH 7.8 and 25 °C. The catalytic efficiency of L1 on this substrate appears therefore to be considerably lower if compared to the action of a typical RIP, such as ricin A chain, on intact rat ribosomes which has been reported to occur with a Km of 2.6 µM and a kcat of 1777 min^−1^ [29]. The biological significance of the activity against rRNA at sites different from SRL, and on substrates other than the ribosomes (DNA, viral RNA, poly(A) mRNA) remains to be firmly established. When rRNA was extracted from mammalian cells exposed either to seed saporin or ricin, rRNA was found to be depurinated at a single site presumably corresponding to the one targeted by ricin [30]. Analysis of the in vivo activity of L1/L3 saporin will be required to assess whether multiple depurination plays any major role in the intoxication process and to clarify the mechanism of 80S ribosome inactivation by L1. The strong spatial similarities between type I RIPs, as shown in Figure 3, might suggest that different specificities/enzymatic activities could only reside in a restricted polypeptide area while assessment of whether these regions may contribute to altered activity would require either site-directed mutagenesis or protein domain swapping experiments.

Native ricin A chain carries two N-glycosylation (Asn-X-Ser/Thr) sites and is very poor in lysines (only two residues), a feature that has been linked to the cytosolic entry route of ricin A chains (see Section 5) [32]. Mature saporin has no oligosaccharide side chains similar to most type I RIPs that are hypothesized to be internalized by animal cells mainly passively by fluid-phase pinocytosis, with some relevant exceptions (see Section 5). Recently, a variant PAP from asiatic *Phytolacca acinosa*, PAP-S1aci, was resolved at 1.7 Angstroms (sharing 95% identity with PAP-S, one seed isoform of *Phytolacca americana*) and was found to have a proline substitution in the active site (Pro174) together with a rare type of N-glycosylation consisting of N-acetyl-D-glucosamine residues linked to the Asn10- Asn44- Asn255 sites, corresponding to putative rRNA-binding regions, mapped through computer modelling studies based on their structural data [33]. The authors hypothesize that these GlcNAc modifications may have evolved to exploit Mannose/GlcNAc-receptor-mediated endocytosis to enhance cytotoxic activity against seed predators. Their computer simulation studies suggest that PAP-S1aci depurination activity would be adversely affected by larger, more typical oligosaccharide chains. The presence of short mannosyl residues in PAP-S1aci may thus confer an advantage to these seeds without compromising their RIP catalytic activity. The absence of carbohydrate side chains on PAP-I, a cell-wall protein may reflect its specialized antiviral role for “local suicide” of the virus- infected cells (see below).

## 3. Biotechnological Application in Agriculture

To explore the potential antimicrobial activity, different RIPs, including pokeweed antiviral protein (PAP), trichosanthin (TC) from *Trichosanthes kirilowii* Maxim and the antiviral protein from *Phytolacca insularis* Nakai have been expressed in transgenic plants successfully leading to plant resistance against various viral and/or fungal proteins [34,35,36,37]. Transformed tobacco plants with the nontoxic C-terminal deletion mutant PAPW237* of *Phytolacca americana* and the active site mutant PAPE176V showed both that the ribosomes from these transgenic tobacco plants were not found depurinated. Interestingly, extracts from transgenic plants expressing PAPW237* protected tobacco plants from Potato Virus X infection, while the plant extracts from catalytic site mutant PAPE176V did not. PAP proteins have been studied since the nineties by the group headed by Tumer focusing on their potential applications to agriculture [38]. They also found that transgenic *Arabidopsis* (Line 512) plant expressing PAPW237* displayed an enhanced resistance to a strain of Tobacco Etch virus (TEV) and detected several genes upregulated, including auxin-responsive genes (more than 4-fold vs. basal), as well as genes involved in immunity and plant defense when performing a transcript profiling analysis using the Affymetrix *Arabidopsis* microarray. Ribosomal RNA is not the only substrate for PAP enzymatic activity, and capped mRNAs and uncapped RNAs are subject to inactivation by PAP. Additionally, double-stranded (ds) supercoiled DNA could be cleaved by this toxin [38]. The catalytic site required for rRNA depurination is the same required for DNA cleavage, as the PAPE176V mutant also could not cleave dsDNA, which, when treated with PAPwt, contained apurinic/apyrimidinic (AP) sites due to the removal of adenines. This same PAG activity has also been reported for different other type I RIPs including Gelonin, momordin I, PAP-S and saporin [26], but not for the ricin A chain. However, its role in the intoxication process by RIPs has yet to be elucidated.

Due to the observation that several monomeric RIPs are active also towards “conspecific” ribosomes, and, because of this observation, inefficient endoplasmic reticulum (ER) translocation or mistargeting of RIPs potentially can lead to cell self-intoxication. Cells would need to prevent any unregulated accumulation of the catalytically active enzymes in the cytosolic compartment, implying that this class of enzymes could block the spread of certain pathogens by causing local suicide of the infected cells [39,40]. Following this hypothesis [41], plant cells undergoing plasma membrane breaching by a virus would allow entry of apoplast-located toxin(s). This localized cell death would concomitantly block viral replication and thus eventually block the systemic spread of the virus load throughout the plant. Although this has not always been the case, as when the Iris RIPs were expressed in transgenic tobacco plants [42], where only a local protection could be observed. A recent study showed that PAP formed homodimer complexes in the cytosol of pokeweed cells while its monomeric form was instead found in the apoplast [43]. PAP homodimers were much less active on rRNA as compared to the monomeric PAP. Thus, at least in the case of PAP, homodimerization is another mechanism to avoid this plant RIP depurinating its own rRNA. Since plants are often infected with multiple pathogens, it was suggested that engineering them with single RIP genes, such as the nontoxic PAP variant, which leaves intact host ribosomes and confer broad-spectrum disease resistance may be an advantageous application in agriculture to pursue.

## 4. ER-Stress Mediated Regulation of Type I RIPs’ Signal Peptides

When heterologously expressed in tobacco protoplasts in the absence of the B chain, the catalytically active A chain of ricin is retained in the ER and then retrotranslocated to the cytosol where it inactivates tobacco ribosomes [6]. PAP has been suggested to follow a similar intoxication pathway when heterologously expressed in yeast cells [38]. To determine whether saporin expression was toxic to tobacco protoplasts, Marshall and colleagues used the sequence encoding the saporin precursor to study its fate in leaf protoplasts [16]. The experiments were performed using initially the non-toxic preproSAPKQ mutant, encoding 24 amino acid saporin signal peptide, 253-amino acid mature seed sequence isoform, and the C-terminal propeptide. In contrast to what observed with ricin A chain, inactive saporin polypeptides were found to be efficiently secreted into the incubation media [16]. Saporin wild type expression was, nevertheless, highly toxic to the protoplasts, indicating the newly synthesized polypeptides could have reached the cytosol (i) either because a few failed to be targeted to the ER or (ii) because they could retrotranslocate, as ricin A chain and PAP, or (iii) lastly, because a pool of endocytosed molecules would intoxicate them. However, when control protoplasts were co-incubated with saporin-expressing protoplasts, toxicity was found to be endogenously produced [16]. Mutated saporin variants at the catalytic site with an intermediate reduction in RIP activity were used, one termed SAPV (having Valine176 instead of Glutamic acid), as to still allow detection of saporin polypeptide biosynthesis. When specific mutations were introduced which abolished signal peptide cleavage in this background, considerably more mutant preproSAPV was recovered from tobacco protoplasts (as compared to preproSAPV) with their experiments confirming that when the former was co-expressed with a phaseolin reporter, toxicity was consistently much lower [16]. Saporin cytotoxicity apparently involved the action of toxin molecules that were at least first partially inserted into ER membranes. Retrotranslocation of ricin A chain from the ER to the cytosol has been shown to be dependent on the activity of an AAA-ATPase known as CDC48 in plant and yeast cells (p97 in mammalian cells) [44]. They thus further investigated whether saporin toxicity would also be dependent on CDC48 extraction. Unexpectedly, co-expression of dominant negative CDC48 QQ mutant, exacerbated saporin toxicity, as shown by an over 80% reduction in phaseolin reporter biosynthesis [16]. These observations were totally opposite to the effects of CDC48 QQ expression on RTA toxicity, where, on the contrary, a significant rescue in protein synthesis is observed [44]. Expression of CDC48 QQ in tobacco protoplasts causes an upregulation of several ER chaperones, including the Binding Immunoglobulin Protein (BiP), Calreticulin and Endoplasmin [44], due to induction of the unfolded protein response, caused by a disregulated accumulation of misfolded or orphan proteins [45]. ER stress could also be induced via treatment with the N-glycosylation inhibitor Tunicamycin showing saporin toxicity increased similarly to what seen upon co-expression with CDC48 QQ [16]. This observation was reminiscent of the “pre-emptive quality control pathway” a novel mechanism by which subcellular protein localization can be regulated in response to stress, as reported for the mammalian prion protein (PrP) [46,47]. The pathway was identified based on the observation of ER translocation of chaperones such as protein disulfide isomerase (PDI) or BiP, a critical facet of the ER stress response, is not attenuated even during maximal acute stress [46] and depends upon characteristics of the chaperone’s signal peptide. Targeting of the saporin precursor to the ER carrying instead tobacco BiP’s signal peptide prevented cytotoxicity, as expected [16]. This mechanism of toxicity modulation upon ER stress may have further implications for the role of type I RIPs in plant defense against pathogen attack.

**Figure 4:**
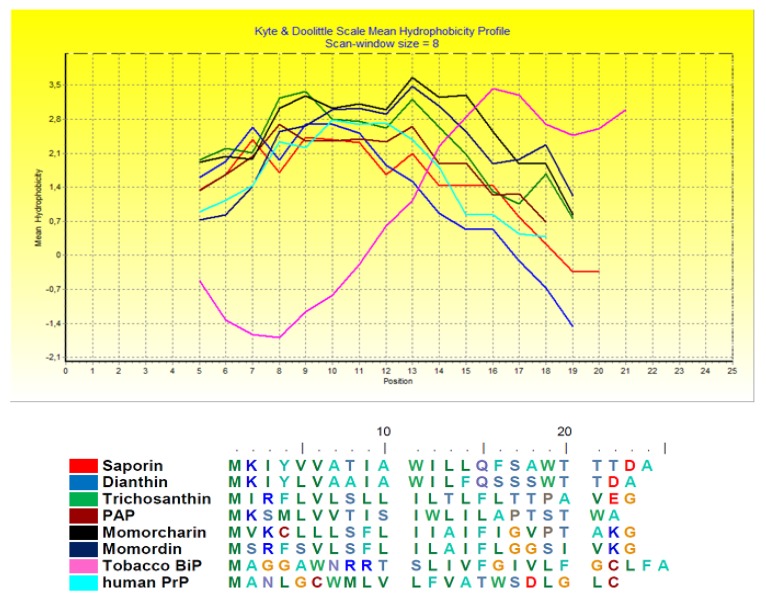
Kyte–Doolittle hydrophobicity Plot of the signal peptides of type I RIPs, tobacco BiP and PrP protein obtained by BioEdit software (version 7.0.9.0, Ibis Therapeutics, Carlsbad, CA, USA). Sequences used are shown under the plots.

Here, we wonder if other type I plant RIPs may undergo such an ER-stress response and show for a comparison the hydrophobicity plot of signal peptides of the chaperone BiP with those of PrP, saporin, dianthin, trichosanthin PAP, momorcharin and momordin (Figure 4).

**Figure 5:**
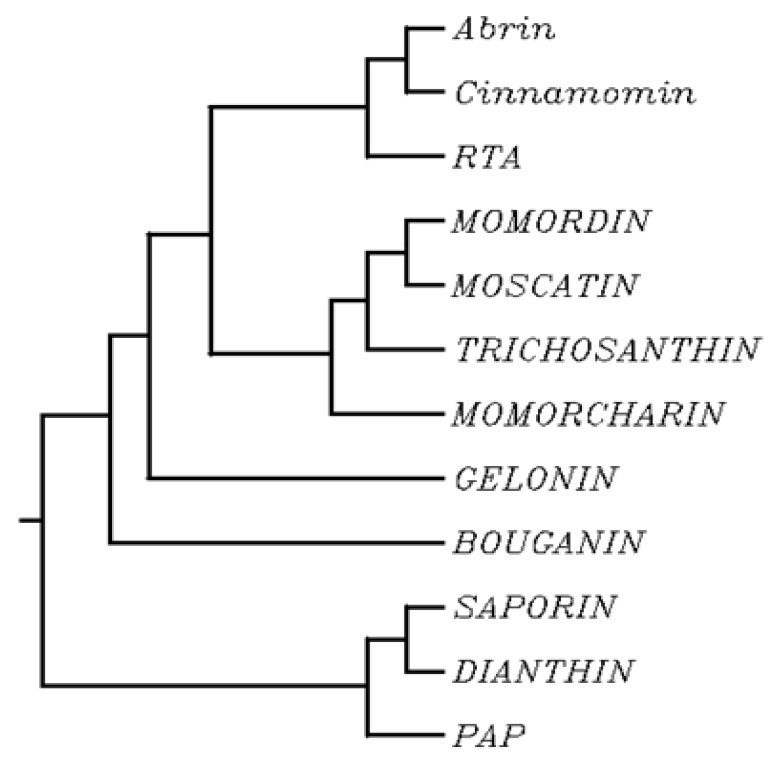
Phylogenetic tree of type I and type II RIPs obtained using the amino acidic sequences aligned with the Clustal Omega on-line software (EMBL-EBI, Hinxton, UK) with standard parameters. The branch length represents the number of changes that occurred in that branch.

Comparing the hydrophobicity plot of saporin and a few other type I RIP signal peptides indicate that they are similar in distribution to the signal peptide of Prion protein (PrP). At least for these six type I RIPs, the behavior in response to ER-stress might be expected to overlap with the one described for PrP and saporin. In contrast, Gelonin signal peptide displays a quite different behavior and is not expected to undergo this ER-stress regulated pre-emptive pathway (not shown). Interestingly, if we look at the phylogenetic tree of some type I (and few type II RIPs) (Figure 5), it shows that Gelonin, as well as bouganin constitute independent branches of this tree when compared to the other type I RIPs investigated here that can be found grouped into two subgroups one constituted by saporin dianthin and PAP and the second one including trichosanthin, momordin and momorcharin.

## 5. Intoxication Routes in Mammalian Cells

**Figure 6:**
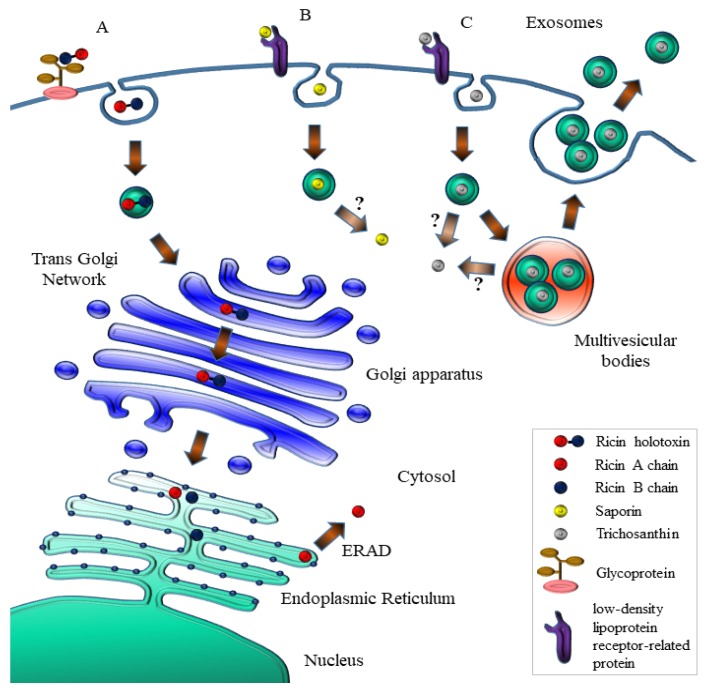
Schematic representation of the intoxication routes followed by ricin (**A**); saporin (**B**) and trichosanthin (**C**), modified from [56].

Ricin holotoxin, as other type II RIPs, is able to exploit multiple receptors to enter mammalian cells thanks to the B-chain lectin properties binding cell surface galactoses/N-acetylgalactosamines and internalizing by (multiple) receptor-mediated endocytosis. Most of the holotoxin is then either recycled back to the cell surface or transported to endosomes where it is in part able to avoid proteolytic degradation in the endo-lysosomal compartment [48], while few molecules undergo retrograde transport reaching the Trans Golgi network (TGN) [49] and passing through the Golgi cisternae, where it was demonstrated that a modified RTA chain containing sulfation sites could be, indeed, found sulfated [50], to finally reach the ER, presumably thanks to interactions with recycling chaperones (calnexin/calreticulin) where the catalytic A chain (RTA) must be reductively separated from the cell-binding B chain [51] thanks to the action of PDI and thioredoxin-reductases [52,53]. Free RTA then mimics as a misfolded protein and is targeted to proteasomal degradation [48,54,55] by interacting with EDEM-1 (ER degradation-enhancing α-mannosidase I-like protein 1) and EDEM-2, which are key players in redirecting aberrant proteins for the ER-associated protein degradation (ERAD) (Figure 6).

Both were shown to interact with ricin [57], but EDEM-2 may promote retrotranslocation out of the ER independently of translocon accessibility. Co-immunoprecipitation experiments demonstrated that ricin A chain interacted preferentially with EDEM-2. Ricin A chain used for these experiments does not contain carbohydrates (recombinantly expressed in *E. coli*) and therefore ricin interaction with the two EDEM proteins, in these experiments, represents nonglycan-mediated interplays. A single point mutation in ricin A-chain increases toxin degradation and inhibits EDEM-1-dependent ER retrotranslocation [58]. RTA contains a 12-residue hydrophobic C-terminal region that becomes exposed after the reduction of ricin in the ER. Indeed, mutation P250A increases also the endo-lysosomal degradation of the toxin, as well as reduces its transport out of the ER into the cytosol. The retrotranslocated RTA chain reaches the cytosol where it may refold rapidly, facilitated also by interaction with the target ribosomes [55]. RTA mimics ERAD substrates, escaping proteasomal degradation presumably also due to its paucity in lysine residues [32] and once in the cytosol, RTA interacts with Hsc70 chaperones, with its final destiny (refolding or degradation) depending on the presence of the co-chaperones that regulate Hsc70 activity [59]. Recently, with the aim of neutralizing ricin cytoxicity (which is an intensive field of investigation, see below) in ricin challenged mice they injected JJX12 a camelid bispecific antibody recognizing both an epitope on RTA (VH) and another one (VL) on the galactose-binding subunit that could passively protect mice against holotoxin lethal doses. JJX12 could affect dynamics of ricin uptake and trafficking also in human epithelial cells. Confocal microscopy and live cell imaging revealed that JJX12-toxin complexes formed at the cell surface could internalize via a pathway sensitive to amiloride, a known inhibitor of macropinocytosis. JJX12 interfered with retrograde transport of ricin to the TGN with the accumulation of the toxin in late endosomes found significantly enhanced [60].

The internalization pathway followed by type I RIPs in mammalian cells deserved much less attention, since it has been assumed that it should not rely on receptor-mediated endocytosis. Saporin cytotoxicity varies dramatically between different mammalian cell lines, with concentrations inhibiting protein synthesis by 50% (IC_50_) ranging from the subnanomolar to the micromolar range, spanning at least three orders of magnitude [18,61]. In the case of saporin and trichosanthin, a family of closely related receptors has been identified to be involved in their internalization: the low-density lipoprotein receptor (LDLR) family that includes seven closely related family members: the very-low-density lipoprotein (VLDL) receptor, apoE receptor 2, multiple epidermal growth factor-like domains 7 (MEGF7), glycoprotein 330 (gp330/megalin/LRP2), lipoprotein receptor-related protein 1 (LRP1), and LRP1B. These proteins were shown to be promiscuous in ligand binding [62]. The α2-macroglobulin receptor/low-density lipoprotein receptor-related protein (LRP1) was shown to bind saporin in vitro [61,63] mediating saporin internalization in human monocytes and fibroblasts [64,65] (Figure 6). In human promyelocytic U937 cells, downregulation of LRP nicely paralleled resistance to saporin and to a urokinase–saporin conjugate [64]. Specific displacement of iodinated LRP1-receptor associated protein (RAP) with saporin in these cells was also independently demonstrated [66]. Mouse embryonic fibroblasts (MEF-2) derived from LRP1 knock-out mice were found 10-fold less sensitive to saporin as compared to MEF-1 control cells carrying both LRP1 and low-density lipoprotein receptor (LDLR) [30]. LB6 fibroblasts transfected with the human receptor for urokinase were used to study internalization of a human urokinase–saporin conjugate, which was initially designed to be internalized only following interaction of the urokinase domain with plasminogen activator inhibitors: surprisingly, saporin domain was instead able to trigger internalization of this conjugate. Confocal studies demonstrated a clear role for LRP1 in saporin–conjugate internalization [67]. Previously, a recombinant chimeric fusion in which only the amino-terminal fragment of human urokinase (unable to mediate internalization) was fused to saporin was found to be efficiently endocytosed by target cells [63]. While LRP1 is clearly involved in saporin uptake, at least in different cell lines, it did not appear to be essential for saporin cytotoxicity in Chinese hamster ovary (CHO) mutant cell line, CHO 13-5-1 [68], which had no detectable LRP mRNA. However, CHO cell lines defective for expression of either heparan sulphates or proteoglycans were also shown to be as sensitive to saporin isoforms as the parental CHO cells [65], suggesting that multiple receptors may be exploited by saporin in CHO. Trichosanthin (TC) is known to behave as an invasive toxin that targets syncythiotrophoblasts, macrophages, and T-cells and whose uptake was recently investigated [69]. TC binds cell surface receptors belonging to the LDL-related receptor family (Figure 6), and its well-known abortifaciens and renotoxic actions are caused presumably by LRP1-mediated uptake in trophoblasts and by LRP2/megalin-mediated uptake in proximal tubule epithelial cells [70]. Jurkatt-T cells, which do not express members of the LDL receptor family, are essentially resistant to free TC (as they are to saporin, our unpublished results), but turned extremely sensitive to the TC-loaded vesicles, which were secreted by at least two different target cell lines, JAR and K562, where part of the endocytosed TC was found incorporated into “pomegrenade” vesicles, deriving from multivesicular body (MVB) membranes, being subsequently secreted upon fusion of the MVB with the plasma membrane (Figure 6), thus targeting both syngeneic and allogeneic cells (Jurkatt-T cells). However, the possibility that other type I RIPs could exploit this exosome-mediated intercellular trafficking route remains to be investigated. Interestingly, Gelonin chimeric fusions were shown to be able to trigger mammalian cell death following instead of usual apoptotic an autophagy pathway [71,72].

The intracellular site(s) from which type I RIPs can escape into the cytosol remain unknown. However, several lines of evidence would exclude that the ricin Golgi-mediated retrograde transport to the ER could be used by other type I plant RIPs to exert their cytotoxicity. Brefeldin A, a fungal metabolite that causes disassembly of the Golgi complex, completely blocks ricin and RTA cytotoxicity [73,74], but fails to reduce saporin toxicity [30]. KDEL (single amino acid letter code) ER-retrieval sequence appended to saporin did not enhance saporin cytotoxicity [30], in contrast to its potentiating effects on RTA cytotoxicity [75]. ERAD mutants of CHO cells found resistant to ricin and *Pseudomonas* exotoxin A (PEA) remained as sensitive to saporin as parental CHO cells [76,77]. In addition, other biochemical essential features of RTA are not shared with some other type I RIPs. Lipid partitioning studies using Triton X-114 demonstrated that while RTA is predominantly found in the detergent phase, the ricin B chain, ricin holotoxin and several type I RIPs, including saporin, are instead found in the aqueous phase [78]. Most importantly, RTA has been shown to interact with negatively charged lipid vesicles and with ER membranes, undergoing a conformational change that makes it a better substrate for the ERAD system [79]. Saporin, in contrast, is a very stable protein, does not stably associate with negatively charged vesicles [32,65,79,80] and undergoes a peculiar stress-induced cytosolic delivery [16]. Altogether, the available data in literature would indicate that RTA compared to type I RIPs, such as saporin, may exploit different strategies to reach the cytosolic compartment in intoxicated cells.

## 6. Cell Death and Intracellular Signaling

Initially, it was assumed that mammalian cells exposed to the plant toxins would die simply because of the blockade of their protein translation machinery. However, it was shortly clear that besides inhibition of translation other players were to be involved in mediating cell-death. Griffiths and colleagues showed that intramuscular injection of ricin or abrin into rats resulted in the death of cells in rapidly dividing tissues, where the toxins were more concentrated. They observed that the morphology of dying cells in para-aortic lymph nodes and Peyers patches both in ricin and abrin injected-rats closely resembled that in cells undergoing apoptotic cell death [81,82].

Caspases are among the principal effectors of apoptosis, involved in pathways such as caspase-8-regulated extrinsic and caspase-9-regulated intrinsic pathways. The caspase-9 pathway links mitochondrial damage to caspase activation, and serves as an index of damage for the mitochondrial membrane function that loses its membrane potential [83]. Furthermore, co-downstream member caspase-3 is an executor of the DNA fragmentation and final steps leading to apoptosis, as exemplified by cleavage of poly(ADP-ribose) polymerase (PARP) during cell death [84].

Apoptotic death of U937 cells by ricin was evidenced by nuclear morphological changes, DNA fragmentation and an increased caspase-like activity. Komatsu and colleagues found also that an early event was that intracellular NAD(+) and ATP levels decreased in ricin-treated U937 cells, followed by the well-known ricin-mediated protein synthesis inhibition. The PARP inhibitor, 3-aminobenzamide (3-ABA), prevented depletion in NAD(+) and ATP levels, also protecting U937 cells from lysis. Overall, their results indicated that multiple apoptotic signaling pathways were triggered by ricin-treatment, one pathway leading to cell lysis via PARP activation while the other one lead to DNA fragmentation and caspase activation [85]. Toxicity with respect to apoptosis induction by various RIPs and the low molecular weight protein synthesis inhibitor, cycloheximide (CHX) was compared among various adherent and non-adherent cell lines, in particular focusing on the type II RIP abrin in one study [86]. Although there were no major differences observed between IC_50_s for protein synthesis inhibition in Jurkat-T cells or U937 cells using abrin, the extent of apoptosis was found to differ significantly. Most of the signaling cascades and pathways triggered by RIPs that are leading to apoptotic cell death were found funneled via mitochondria that are, indeed, major players in several stress-induced cell death pathways. Iordanov et al. show that ricin, α-sarcin and anisomycin were able to activate the SAP kinases or JNK1 in Rat-1 cells [87]. The damage to 28S rRNA by RIPs leads to a novel pathway of kinase activation known as “ribotoxic stress”. Several type I RIPs, including TC, Gelonin and saporin have been demonstrated to activate these apoptotic pathways. In the latter case, the authors also suggested that initiation of apoptosis through mitochondrial cascade in U937 cells was an earlier event than the inhibition of translation by the plant RIP [88].

Another possible mode of action by plant toxins involves Reactive Oxygen Species (ROS) production, in response to stress and increase in intracellular calcium levels. Abrin was able to induce apoptosis by production of ROS, loss of mitochondrial membrane potential and release of cytochrome c into the cytosol. Shih et al. proposed that the apoptogenic activity of abrin A-chain also could be independent of its RNA N-glycosidase activity [86]. However, recently, a 225-fold lower abrin active-site mutant rABRA (R167L) was conjugated to the ricin B chain and its effects compared to a hybrid ricin B chain- abrin-conjugate in Jurkat-T cells. Rate of inhibition of protein translation, as well as the timing of activation of p38MAPK or caspase-3, were much slower by the mutated ricin B-rABRA (R167L), indicating that the lower abrin RIP potency correlated with the delay in activating cell death pathways, despite both conjugates used the same effectors in mediating cell death [89].

Trichosanthin induced high levels of ROS production in human choriocarcinoma cells [90]. Ricin holotoxin induces a rapid elevation in intracellular calcium levels in hepatoma cells [91]. Liver is one of the target organs for ricin holotoxin metabolism. An interesting study was performed in rats challenged with ricin: by using subcellular fractionation and immunoblotting procedures, the authors followed the fate of ricin in vivo in rat liver, focusing on endosome-associated events and on the induction of apoptosis [92]. Injected ricin rapidly accumulated in endosomes as an intact heterodimer (5 min–90 min) being later found (15 min–90 min) partially translocated to the cytosol as A- (and dissociated B-) chains. Strikingly, holotoxin could be found intact upon incubation with endosomal lysates and even in cell-free endosomes (pre-loaded with ricin in vivo). In the latter, a time-dependent translocation of ricin across the endosomal membrane could be observed depending on thioredoxin reductase-1, which was required to reduce the holotoxin disulphide bridge between A and B chain. Interestingly, ricin B chain alone, as a control, could induce cell death, as well. Holotoxin induced an intrinsic apoptotic pathway with increased cytochrome c release, activation of caspases-9 and -3 and DNA fragmentation. Reduced ricin or ricin B-chain caused cytochrome c release from mitochondria in vivo and in in vitro assays, suggesting ricin B-chain interaction with mitochondria contributes to the holotoxin-induced apoptosis [92].

The overall picture would point to a great level of complexity, suggesting once again that the pathways of apoptosis induced by RIPs are strictly dependent upon the cells lines investigated, as well as from the specific RIP utilized, and should be clarified when choosing to pursue an IT approach. In this context, when analyzing the effects of a targeted toxin, also the choice of the targeting moiety itself may also implicate different/alternate modalities in cell killing capabilities of the IT, as demonstrated by some studies by the group of Bolognesi: they compared cytotoxicities of two immunotoxins one obtained (by chemical conjugation of the plant toxin) conjugating seed saporin to Rituximab, an α-CD20 chimeric humanized antibody already approved by the United States (U.S.) Federal Drug Administration (FDA) to treat lymphomas, and the other one to OM124 an α-CD22 murine antibody. Both ITs were specific against Raji cells, being the α-CD22 murine immunotoxin (IT) two logs more efficient in cell killing Raji cells, presumably due to its faster internalization rate. Moreover, it induced a greater caspase activation compared to Rituximab IT. Cytotoxicity of both ITs could be in part prevented by pan-caspase inhibitor Z-VAD or necrostatin-1 a necroptosis inhibitor. Oxidative stress was involved in the cell-killing activity of Rituximab IT, as demonstrated by the protective role of the hydrogen peroxide scavenger catalase, but not in that of anti-CD22 IT [93]. Thus, depending on the targeting moiety, different pathways may be found activated. On the other hand, another study in L540 human Hodgkin’s lymphoma cells showed at concentrations giving similar cellular inhibition of translation by ricin or saporin that ricin effects were more rapid when compared to those elicited by saporin. In addition, while the intrinsic apoptotic paths were equally activated, ricin activated the extrinsic caspase and effector caspase-3/7 pathways much more efficiently. Overall, the data indicated that different cell death mechanisms could be elicited by ricin and saporin with different timings and relative potency, hence, some of these differences might be also in part attributed to the concomitant pro-apoptotic effect of ricin B chain. The shutdown of intracellular signaling pathways may also contribute to the cytotoxicity of an IT as demonstrated by a recombinant IT made with recombinant Gelonin fused to a humanized single chain antibody (scFv, termed 4D5) deriving from Herceptin an anti Her2/neu [94].

In the case of solid tumors, it has been suggested that higher avidity and longer residence time of IgG-based immunoconjugates would outweigh improved tumor penetration of scFv-based constructs [95]. Since immunoconjugate development has been hampered by nonspecific toxicity and vascular leak syndrome (see below) the group of Rosemblum used Herceptin or its derived humanized single chain antibody (scFv, termed 4D5) to generate a Herceptin/rGel conjugate (bivalent) along with the two corresponding monovalent humanized recombinant ITs obtained using a classic flexible G4S-linker in the two orientations 4D5/rGel or rGel/4D5 [94]. The specific activity of engineered anti-Her2/neu single chain-immunotoxins fused to recombinant Gelonin (rGel) was compared to the activity of bivalent IgG-containing-immunoconjugate. The three constructs despite showing similar affinity to Her2/neu overexpressing ovarian cancer cells demonstrated significantly different antitumor activities. This study confirmed that monovalent fusion constructs can display virtually identical binding affinities as compared to their original IgG-counterpart [94]. However, rGel/4D5 orientation construct and Herceptin/rGel conjugate were found better performing than the 4D5/rGel construct both in in vitro and in vivo efficacy. Herceptin/rGel conjugate showed the most efficient and fastest internalization into target cells. The intracellular release of rGel after endocytosis of the various constructs was assessed after 4h exposure to 25 nM of each construct and the rGel moiety of all the immunotoxins could be observed by Western blots primarily in the cytosolic fraction. The rGel/4D5 displayed a comparatively higher and more prolonged intracellular concentration of toxin than 4D5/rGel. Extremely interesting were the data concerning the Her2/neu-related signaling events in SK-OV-3 cells. Both Herceptin/rGel conjugate and rGel/4D5 showed an impressive inhibition of phosphorylation of Her2/neu, EGFR, Akt and ERK, which are the most critical events for the Her2/neu signaling cascade. In contrast, 4D5/rGel showed a comparatively much reduced effect on these signaling pathways. Thus, the improved cytotoxicity coincided with an increased effect on cell signaling for recombinant rGel/4D5 [94].

## 7. Novel Potential Applications of RIPs

Historically, immunotoxins and chimeric fusions have been developed in parallel, with the former more extensively studied, following the introduction of monoclonal antibodies, which have gained great consideration for their selective specificity, being compared to the Paul Ehrlich’s “magic bullet”. On the other hand, a chimeric fusion Denileukin diftitox is the first, and until now the only, toxin-based formulation approved by the FDA employed in the treatment of cutaneous T-cell lymphomas [96] and tested for other clinical settings. This is a chimeric fusion formed by a truncated form of diphtheria toxin and the human cytokine interleukin-2, produced in bacteria. Toxin-based drugs have been mostly utilized in the treatment of hematological tumors because, being injected in the bloodstream, they can more easily reach target cancer cells. Numerous ITs formed by a toxic RIP domain like saporin, PAP, momordin and RTA, combined with a monoclonal antibody against markers of hematological malignancies such as CD2, CD19, CD22, CD25, CD30, CD38 and CD123 have been tested alone or in combination in animal models of lymphoma and leukemia [97,98,99,100,101,102] and some underwent early clinical phase studies. The anti-cancer activity of several ITs have been assayed also against solid tumors, but did not display the same successful results, likely due to the limited penetration within the tumor mass and relevant immunogenicity, the latter greatly reduced in hematological cancer patients. In the field of ligand-targeted chimerae, toxins have been in particular fused or conjugated to growth factors (EGF, [103], FGF [104] and VEGF [105]), but also with other polypeptides, such as uPa [106] and NCAM [107] as well, whose receptors have been found over-expressed on the cell surface of different cancer cells or in tumor vasculature, allowing to test the same therapeutics on several tumor models. For a comprehensive detailed description of ITs or fusion chimerae made with plant RIPs that have been also assayed using specific enhancers to promote toxin cytosolic delivery, refer to [108]. A historical description of ITs constructed using seed-extracted saporin (also termed saporin-S6) can be found in [109].

**Figure 7:**
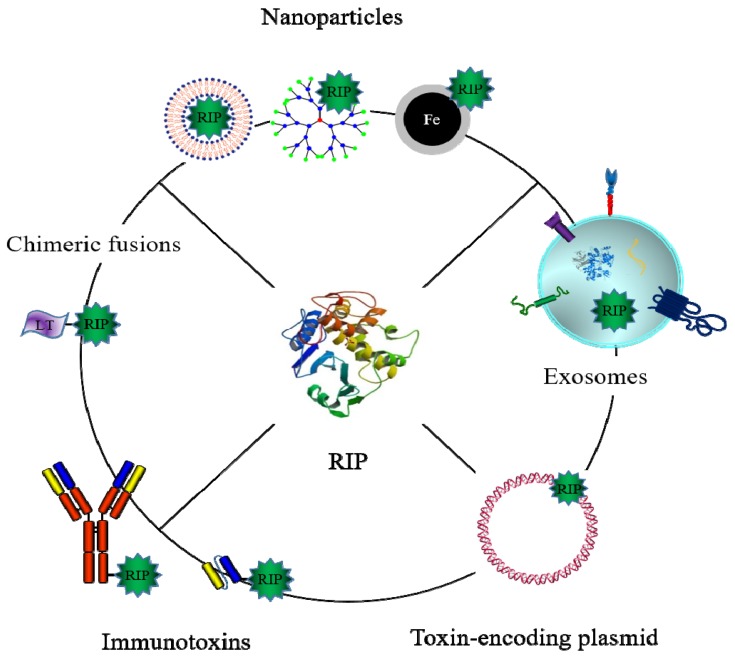
Present and future applications of RIPs. A schematic representation summarizes some of the potential application of a toxic plant RIP (green star and 3D-ribbon) starting with a classical Immunotoxin made by IgG chemically conjugated to native RIPs, which may be further transformed (left) into a fusion recombinant chimera or (right) into single chain variable fusions to RIP or even simply to DNA constructs encoding the plant RIP with all these different molecules being deliverable via targeted exosomes or engineered nanoparticles even for diagnostic purposes with MnFe_2_O_4_ nanoparticles (see text below).

A major drawback of such biotechnological drugs is the triggering of immune responses after repeated administrations. Remarkably, plant toxin-based chimerae show less immunogenicity compared with those of bacterial origin. The development of ITs containing fully humanized antibodies or antibody fragments lacking Fc portions and the de-immunization of the toxin through the substitution of critical reactive amino acid residues contributed to significantly lessen the immunogenicity problems [31]. Furthermore, a serious side effect observed during IT clinical administration is the vascular leak syndrome (VLS), caused by the unspecific binding of the toxin domains to the vascular endothelial cells and characterized by interstitial edema, weight gain, and in most severe cases, pulmonary edema and hypotension. RTA-containing immunotoxins have been reported to elicit VLS [110]. An IT including a RTA mutated in a single amino acid flanking the consensus sequence identified as responsible of VLS exhibited a significantly reduced vascular damage in mouse models [111]. The clinical evaluation of the most promising targeted toxins is ongoing and, by exploiting novel discoveries and technologies in the biomedical fields, new toxin-related therapeutic options could be envisaged. Noteworthy examples are described below and summarized in Figure 7.

### 7.1. Nanoparticles

Important drawbacks in cancer drug delivery approaches are often represented by (i) the scarce aqueous solubility of both natural and synthetic therapeutic agents; (ii) by their rapid clearance; (iii) by the dramatic side effects due to the lack of target selectivity and (iv) by the onset of resistance after repeated treatments.

In the last decades, many efforts have been directed to the development of delivery systems that may modify the drug biodistribution by conferring specific retention in the tumor site and favor tissue uptake. Nanotechnology has demonstrated great potential to overcome these limitations by increasing the circulation time of the drug, helping to bypass the hydrophobic nature of molecules by their incorporation, providing a number of additional administration routes and co-delivery of multiple drugs types and/or diagnostic agents for combined therapies. Furthermore, nanovectors can be arranged for a prompt controlled or sustained release of the drug, reducing the number of administrations and side effects or the risk of resistance [112]. Nanoparticle-based formulations can promote drug release with a selective activation within the target organ/tissue as a result of different stimuli, such as pH, enzymes, heat, light, etc. Liposomes, polymers and inorganic nanoparticles have been successfully employed for the delivery of protein toxins [113,114,115]. A library of cationic lipid-like materials (termed “lipidoids”) was designed and employed to form nanocomplexes with proteins for intracellular delivery. Saporin and RNAse A were used as representative cytotoxic effector proteins, that when complexed with these lipidoids showed efficient cell internalization. A representative lipidoid EC16-1 was selected for protein delivery assay and, when administrated to several cancer cells, saporin was demonstrated to be much more active in all the cell lines tested. Saporin-contained nanoparticles inhibited cell proliferation in vitro and suppressed tumor growth in a murine model of breast cancer [113]. A new transfection reagent, lipofectamine 3000, whose composition has not been displayed, has being tested for enhancing delivery of therapeutic proteins, including saporin [116]. In this recent report, they also show, being surprised that the J774.2 macrophage cell line already exhibits a great sensitivity to saporin, which is most likely due to the presence of LDL related receptor protein (LRP1) at the cell surface of macrophages, mediating uptake of the plant RIP. However, when they use cells that do not expose endocytic receptors for saporin entry, in the presence of lipofectamine 3000 saporin could potently inhibits growth of neuroblastoma N2a cells (EC50 = 1 nM in the presence of LF3000) but not in the presence of other transfection reagents such as lipofectamine LTX or Proteofectene. LF3000 increased the sensitivity of N2a cells to saporin by three orders of magnitude [116]. Stimuli-sensitive nanoconstructs have been investigated to enhance toxin penetration into the cytosol. Nanoparticles formed by polyglutamate (PGA), a sensitive polymer to the digestion by cathepsin B, a metalloproteinase overexpressed in the microenvironment of many tumors, have been loaded with indocyanine green (ICG), a FDA-approved near-infrared (NIR)-absorbing dye, and with saporin. Upon cleavage, ICG and saporin became bioavailable within the tumor context, facilitating their cellular uptake. Following irradiation, light causes endo/lysosome disruption with consequent drug release in the cytosol, where saporin exerts its toxic effect [114]. Another similar approach is the so-called photochemical internalization (PCI) to facilitate protein release to the cytosol after endocytosis of PCI-relevant photosensitizers. Saporin first was coupled to the polyamidoamine (PAMAM) dendrimer to improve its cellular uptake. Then, a further enhancement of the toxic activity was achieved on gingival and nasopharyngeal cancer cell lines after the combination with the PCI technology [115].

A peculiar approach has been tested by Su and collaborators, based on a two-stage delivery, where cancer cells pretreated with a Gelonin-based IT were killed following exposure to endosome-disrupting polymer nanoparticles. Nanoparticles were composed by the pH-sensitive poly(ethyleneoxide)-modified poly(beta-amino ester) (PBAE) encased within a biocompatible phospholipid shell and stabilized by the introduction of a phosphoethanolamine-conjugated polyethylene glycol (PEG) into the lipid coating. The immunotoxin comprised of a fusion of Gelonin to a fibronectin type III binding domain engineered to exhibit high affinity for the well-established cancer-associated EGF receptor. Co-administration allows co-internalization into common endosomes, followed by a swelling due to the “proton sponge effect” and subsequent IT cytosolic delivery. In vitro experiments indicate that such an approach may achieve a highly synergistic enhancement of antitumor activity [117]. The RIP-nanoparticle binomial can be used also as a diagnostic tool. For instance, the chimeric fusion VEGF121/rGel was conjugated to MnFe_2_O_4_ nanoparticles to be employed as a contrast agent, to monitor the efficacy of the antiangiogenic toxin-based therapy by magnetic resonance imaging (MRI). A satisfactory targeting capability to the vascular endothelial growth factor receptor 2 (VEGFR2) over-expressing cells was achieved providing the acquisition of clear neoangiogenic vascular distributions in orthotopic bladder tumor mice, a relevant information for an effective cancer therapy [118]. Despite successful attempts, the exploitation of nanoparticles (NP) for the delivery of toxins is quite underrepresented as compared to other cargos, like siRNAs or small molecules. Still ribosome inactivating proteins are mainly administrated as ITs or chimeric fusions.

Plant RIP-based NP have not only been employed as anti-cancer therapeutics, but also in the therapy of viral diseases, such as the acquired immunodeficiency syndrome (AIDS). An interesting approach to specifically target and kill cells activated early in the process of HIV production was developed. Ricin A chain was encapsulated in a polymer shell to form nanocapsules where a peptide cross-linker cleavable by HIV-1 protease was inserted. Once internalized in HIV infected cells, the cleaved nanocapsules released RTA, shutting down viral and cellular protein synthesis, leading to a prompt death of the producer cells [119].

### 7.2. Natural Vesicle-Mediated Delivery

Not only artificially prepared nanoparticles such as the liposomes as mentioned above, but also naturally produced extracellular vesicles like exosomes have focused attention as one of the next-generation intracellular carriers. Exosomes are secreted extracellular vesicles (diameters of 30–200 nm), containing biofunctional molecules such as microRNAs or enzymes [120,121,122,123,124]. Exosomes participate in cell-to-cell physiologic communication events, but also in disease progressions such as in tumors [120,121,122,123,124], by carrying the encapsulated signaling molecules to other cells/tissues in the body.

Our research group is currently developing saporin-encapsulated exosomes for specific receptor targeting and to promote effective cytosolic delivery by modification of functional peptides on exosomal membranes. Hereafter, we introduce the novel techniques for exosome-based saporin delivery with a detailed clarification of exosomal characteristics and peptide-based techniques.

Because of the pharmaceutical advantages of naturally occurring exosomes such as (1) effective knowledge of cell-to-cell communication extracellular routes; (2) absence of cytotoxicity; (3) controlled immunogenicity; (4) constitutive secretion; (5) encapsulation of additional biofunctional molecules; and (6) co-expression of functional proteins in membranes, they are expected to be next-generation therapeutic carriers [125,126]. Especially, a more sophisticated exosome-based delivery is considered making use of self-exosomes derived from single patients, to prevent any serious immunological-related problem. However, the high abundance of exosomes circulating in serum having negatively charged exosomal membranes [125,127,128,129], which repel negatively charged cellular membranes, would compete for their cellular uptake, and, therefore, methods for increasing cellular exosomal uptake efficacy must be developed to attain most effective intracellular delivery of exosomal contents.

Recently, our research group found that macropinocytosis (accompanied by actin reorganization, ruffling of the plasma membrane, and engulfment of large volumes of extracellular fluid) is an important entry route for the exosome uptake [130]. Stimulation of EGF receptor (EGFR) by exposing the receptor to natural ligands such as EGF induces signal transductions pathways leading to macropinocytosis via activation of the Rho family of small GTPase Rac [131,132]. When A431 cells (human epidermoid carcinoma, high expressor of EGFR) were treated with GFP-tagged exosomes (20 μg/mL) for 24 h at 37 °C, addition of the EGF (500 nM) significantly increased cellular uptake of the exosomes by approximately 27-fold [130]. Treatment with the macropinocytosis inhibitor, 5-(*N*-ethyl-*N*-isopropyl) amirolide (EIPA) instead reduced the internalization efficacy of exosomes. In a similar way, C-X-C chemokine receptor type 4 (CXCR4)-mediated macropinocytosis [133] was stimulated by stromal cell-derived factor 1α (SDF-1α), which enhanced cellular uptake of the exosomes (approximately 2.3-fold by co-treatment of SDF-1α (200 nM)) [130]. These results suggest that activation of the cancer-related receptors may induce an enhanced cellular uptake of exosomes by macropinocytotic entry route. Then, we examined delivery of saporin by encapsulation in exosomes. A431 cells were treated with saporin-encapsulated exosomes (4 μg/mL of the total exosomes, loaded by usage of electroporation) for 48 h at 37 °C, prior to WST-1 assay. Addition of EGF significantly enhanced cytotoxicity of saporin-encapsulated exosomes (in comparison to the absence of EGF), suggesting once again that induction of macropinocytosis by EGF treatment increases cellular uptake of saporin-encapsulated exosomes [130].

We further developed arginine-rich peptide-modified exosomes, which are able to actively induce macropinocytotic cellular uptake [134,135]. Oligoarginine has been shown to induce micropinocytosis via proteoglycans (e.g., syndecan-4) on plasma membranes [136,137]. Oligoarginine peptide (Rn; n = 4–16)-modified exosomes with a cross-linker (N-ε-maleimidocaproyl-oxysulfosuccinimide ester) were prepared by simple mixing of the peptides and target exosomes and the effects of peptide modification on cellular exosome uptake were evaluated [135]. Relative cellular uptake of Rn-conjugated GFP-tagged exosomes (20 μg/mL) was assayed in 10% serum-containing cell culture medium for 24 h at 37 °C into CHO-K1 cells. The modification of R8 peptides on exosomal membranes showed to greatly increase cellular uptake efficacy (29-fold higher than that of intact exosomes). Overall, our results also showed that the number of arginine residues in the peptide sequence modified on the exosomal membranes differently affected the induction of macropinocytosis and cellular uptake efficacy of the exosome. For example, modification of R16 peptides on exosomal membranes resulted in a slightly lower increased cellular uptake efficacy (18-fold higher than that of intact exosomes) [135]. However, when we artificially encapsulated saporin in exosomes (saporin-exosome), and compared to a saporin-exosome with modified R16 peptides, the latter showed higher biological activities than that of R8 peptide [135]. These results suggest that cytosolic release of saporin may differ when delivered by the exosomes.

We also developed a technique to obtain enhanced cytosolic release of exosomal contents in living cells by a simple method, namely using cationic lipids and pH-sensitive fusogenic peptide, GALA (amino acid sequence: WEAALAEALAEALAEHLAEALAEALEALAA) [125]. GALA peptide was designed to mimic viral fusion protein sequences that mediate escape of virus gene from acidic endosomes to cytosol [138]. We first studied the pH-sensitive fusogenic peptide GALA effects to enhance disruption of endosomal/exosomal membranes during endocytosis of exosomes. We already reported that cationic lipids/GALA complex is a useful system for efficient cytosolic delivery of protein loads [139,140]. In the current research, this system was applied to increase both cellular uptake and cytosolic release of exosomes. Cationic lipids were also used as an “adhesive” to paste the GALA peptide, which carries negative charges deriving from the seven glutamic acids [139,140], onto exosomal surface. As a model macromolecule, Texas red-labeled dextran was encapsulated into exosomes by electroporation, while addition of GALA to the complex of exosome and cationic lipids significantly enhanced cytosolic release of dextran inside cells, indicating that GALA may contribute to disruption of both endosomal and exosomal membranes [139,140]. Saporin-encapsulated exosomes in this combinatorial treatment, showed GALA peptide together with cationic lipids increased RIP activity of saporin, which was originally encapsulated in exosomes [125].

By combined usage of biologically functional peptides, we established simple techniques to enhance macropinocytotic uptake and favor cytosolic release of exosomal contents. Due to their pharmaceutical advantages, exosomes are expected to be the next-generation therapeutic carriers, with these experimental approaches being considered to strongly contribute to effective intracellular delivery of toxins in anti-cancer settings.

### 7.3. Suicide Gene Therapy

ITs with chimeric fusions represent the most diffused attempts to develop toxin-based targeted therapeutics. They display all major drawbacks common to proteinaceous biotechnological drugs, such as costly and time-consuming production and purification while potentially triggering immune responses. In addition, their cytotoxicity is linked to multiple parameters, such as accessibility and penetration inside the tumor mass, specific internalization into target cells, followed by an efficient release of the toxin in the cytoplasm, where it exerts the catalytic activity. Binding and internalization of the IT or chimeric toxin can be blocked through the loss or downregulation of targeted specific receptors by tumor cells, especially after repeated administrations [141]. To overcome some of these limitations, suicide gene therapy has been proposed as an alternative, making use of nucleic acids encoding the toxin domain under control of specific promoters. DNA holds several advantages: its production and management are cheaper and less time-consuming, as compared to manufacturing proteins; low doses are immediately effective, since few molecules produced in the cytosolic compartment are sufficient to achieve the killing activity. DNA-based therapeutics is both less immunogenic and less prone to induce drug resistance. The expression of the effector protein can be tightly controlled by inserting in the construct selected regulatory elements, like inducible or tumor-specific promoters to prevent detrimental expression of the toxin within healthy tissues. The most challenging step in the suicide gene therapy concerns an efficient and safe delivery of the DNA to cancer cells, and two main strategies have been developed for this purpose. Recombinant disarmed viruses or non-viral vectors have been investigated and both display some advantages and constrains. As long as an ideal system for all purposes has not been identified yet, the employment of the right vector has to be established according to the specific characteristics of the disease to be treated. To date, only a few toxins have been studied for cancer suicide gene therapy approaches with diphtheria toxin (DT) being considered a breakthrough, as it has been shown to be effective in numerous cellular and animal models [142]. Two phase I/II clinical trials have been performed where the DT expression was regulated by the H19 promoter, a well-known tumor-associated gene. The H19-DT construct was complexed with the synthetic polycation polyethylenimine (PEI) and administrated intratumorally in patients with recurrent, multiple non muscle invasive bladder tumors, resulting in a prolonged time to recurrence in responding patients (64% at 3 months, 40% at 2 years) [143]. Concerning plant RIPs, a direct intratumoral injection of a saporin-encoding plasmid under cytomegalovirus promoter complexed with the cationic lipid DOTAP in a melanoma mouse model determined a significant reduction of tumor growth with the antitumor effect being enhanced by repeated administrations. By replacing two key amino acids essential for the saporin catalytic activity (SAPKQ mutant), the anti-tumor effect was abolished, demonstrating that it was specifically due to the RIP N-glycosidase activity [14,144]. DNA constructs expressing ricin A chain or *Pseudomonas aeruginosa* exotoxin A genes under the control of the thyroid hormone promoter were delivered by retroviral vectors to glioblastoma cells and were able to eradicate brain tumors in rats [145]. So far, the therapeutic use of cationic lipid/polymers was mainly limited to intratumoral injections because their delivery capacity is prominently hampered by serum components and systemic toxicity occurs. Therefore, this approach is mostly confined to accessible tumors whose surface antigen markers have not been identified yet. Toxin-mediated gene therapy can be used to reduce tumor mass and allow surgery to better eradicate it, or to kill residual tumor cells after tumor surgery. To surmount these restrictions and extend the application of such an approach, masking agents like PEG were included in these formulations, increasing the circulation time in the bloodstream and reducing liposome toxicity. The selectivity towards target cells of toxin DNA constructs could be improved by concomitant association of antibodies or peptides. For instance, basic fibroblast growth factor 2, modified to carry a lysine stretch, was shown to effectively bind plasmid DNA encoding saporin and to deliver it to fibroblast growth factor receptor (FGFR) bearing cells, leading to cell death [146].

By encapsulating a DT-expressing plasmid in poly (lactic-co-glycolic-acid) (PLGA) nanoparticles modified with the carboxy-terminal–binding domain of the *Clostridium perfringens* enterotoxin (CPE), the authors could confer them specificity towards the claudin-3/4 expressing cells [147]. The tumor-specific expression of DT was warranted by the transcriptional control of the p16 promoter. P16 protein is overexpressed in the majority of ovarian cancers correlating with tumor progression and poor prognosis. Nanoparticles were tested in chemotherapy-resistant ovarian cancer cells and within 12 h after intraperitoneal injection in mice harboring ovarian cancer xenografts, they caused a significant inhibition of tumor growth [147]. This is an approach coping therapeutic efficacy with a high safety profile, and could easily be extended to other tumors overexpressing claudin-3/4, such as pancreatic, breast, and prostate cancers. This represents a considerable step forward in the toxin-based suicide gene therapy for its high versatility.

### 7.4. Vaccines

Since Georgi Markov, the Bulgarian novelist and playwright, dissident of the communist regime was killed in London with a ricin pellet shot by a specially modified umbrella in the 1978 [148], type II RIPs have gained widespread attention as potential chemical weapons. Ricin is one of the deadliest poisons in nature; just a single ricin molecule entering the cytosol inactivates over 1.500 ribosomes per minute, causing immediate cell death whereas as little as 500 µg ricin may kill an adult. Initial symptoms in humans may occur within 6–8 h of exposure and clinical symptoms typically progress over 4 to 36 h. Death from ricin poisoning takes place within 36 to 72 h of exposure, depending on the route of exposure and the dose received [149]. People could be exposed to it through the air, food or water. It is unlikely to be absorbed through intact skin; however, the contact with ricin powder or ricin-based products may cause severe irritation and pain [149]. In the 1940s, ricin had been experimented with by U.S. military as a possible warfare agent and was reported in the 1980s as being a warfare agent used in Iraq. In November 2003, the U.S. Secret Service intercepted a letter addressed to the President and delivered to the White House, after it was found to contain ricin. Ten years later, an envelope that preliminarily tested positive for ricin has been again delivered to an U.S. President. Therefore, the need to quickly detect ricin in environmental samples, beverages or food matrices and especially to be able to neutralize or prevent its lethal effects in the case of a biological threat attack has become more and more urgent. Several technologies have been developed so far to allow for sensitive and rapid ricin detection assays. Radioimmunoassay, enhanced colorimetric and chemiluminescence ELISA, immuno-polymerase chain reaction, capillary electrophoresis/liquid chromatography coupled with mass spectrometry, array biosensors, microfluid chip-based immunoassay have been successfully tested on minute amount of ricin, showing sensitivity to power detection even at picomolar levels [149,150]. Medical treatment of patients after ricin exposures is largely symptomatic and currently there is no available antidote. Upon ricin inhalation, the likely symptoms are respiratory distress, fever, nausea, and tightness in the chest, followed by pulmonary edema, low blood pressure and respiratory failure, leading to death. The treatment is based on oxygen administration, bronchodilators, endotracheal intubation and supplemental positive end-expiratory pressure [149]. Significant efforts to develop a recombinant anti-ricin vaccine, as a countermeasure prevention of a biological attack, have been performed, ricin being classified as a biological threat by the U.S. Centers for Disease Control and Prevention. Therefore, several Phase I clinical trials have been launched [151] with two vaccines: RiVax™ and RVEc™. RiVax consists of a recombinant RTA carrying two mutations (V76M, Y80A) not affecting the tertiary structure of the protein, which abrogate both the enzymatic activity and the ability to induce vascular leak syndrome in humans. The protein has been expressed in bacteria and is immunogenic and not toxic in mice, rabbits and humans [152]. In a study on Rhesus Macaques, the parenteral immunization with RiVax elicited a serum antibody response that completely protected them from a lethal dose of aerosolized ricin [153]. The second vaccine candidate, RVEc^TM^, has been developed by investigators at the United States Army Medical Research Institute of Infectious Diseases (USAMRIID) and consists in a truncated version of RTA lacking the C-terminal 68 residues as well as a small hydrophobic loop in the N-terminus, containing a potential protease-sensitive site on the protein surface [154]. Despite deletions, RVEc’s 3D structure is superimposable onto RTA and it also elicits protective antibodies in rodents, New Zealand white rabbits, and African green monkeys [154,155,156,157]. A pilot clinical trial in humans has been carried with RiVax, by injecting three groups of five normal volunteers three times, at monthly intervals with 10, 33, or 100 µg of the vaccine. Ricin neutralizing antibodies were elicited in 1/5 individuals in the low-dose group, 4/5 in the intermediate-dose group, and in all in the high-dose group. Anti-RTA IgGs purified from pools of positive sera were effective in protecting mice from ricin intoxication. Side effects were limited in seven individuals to a modest increase in creatine phosphokinase (CPK) enzyme that was possibly correlated to the intramuscular (i.m.) injection, and a significant increase in only one individual. Nevertheless, the duration of the antibody responses was limited, spanning from 14 to 127 days after the third vaccination [152]. A second pilot phase I, dose escalation clinical trial using RiVax/alum, based on the results obtained on mice showing an enhanced response by approximately 10-fold and protective for at least a year [158,159]. The good manufacturing practice (GMP)-produced vaccine was adsorbed to aluminum salt adjuvant and three dose levels (1, 10 or 100 µg) were administrated to four or five volunteers per group. RiVax/alum induced higher titers of both total and neutralizing antibodies and in some volunteers, the titers were long lasting (five of eight were positive after one year). All volunteers experienced one or more toxicities associated with the i.m. injection [159]. The next steps will include tests on non-human primates and larger clinical trials to confirm the induction of a longer-lasting response. Concomitantly, a phase 1 escalating, multiple-dose study evaluating the safety and immunogenicity of RVEc^TM^ in healthy adults was performed showing that the vaccine was well tolerated and immunogenic at 20 and 50 µg dose levels. Significant ricin-specific ELISA antibody levels were seen in 100% of volunteers after the third dose [157]. The frequencies and types of adverse events experienced after RVEc^TM^ administration were similar to those reported for RiVax^TM^ vaccine, likely reflecting muscle damage associated to i.m. injections, as also reported for other approved vaccines [157]. Two phase I clinical trials are currently withdrawing volunteers (NCT02385825 and NCT02386150) to determine the safety and immunogenicity of a series of three primary vaccinations and a booster vaccination of RVEC^TM^, administered at 10, 50, or 75 μg intramuscularly or at 10 or 20 μg intradermally to healthy adults. These studies are aimed at evaluating if the vaccine will display an acceptable safety profile and if it will elicit anti-ricin antibodies and ricin toxin-neutralizing antibodies in vaccine recipients.

One of the major hurdles to evaluating the vaccine efficacy in human beings is represented by the fact that the titer of ricin-specific serum antibodies, as well as the toxin-neutralizing antibody levels, not being accepted as definitive predictors of protective immunity [160].

### 7.5. Strategies to Promote Endosomal Escape

The number of targeted toxins under investigation in the tumor treatment has considerably risen over time, but in spite of their elevated toxicity, their efficacy in animal models remains far below expectations—a major reason being their limited reaching of cytosolic target ribosomes, due to either cell surface recycling after receptor-mediated internalization or to proteolytic degradation in lysosomes. Several strategies have been developed to improve the toxin performance by facilitating their release into the cytosol, where the toxic activity occurs. Different chemicals, cell-penetrating or fusogenic peptides and light-inducible techniques have been explored to destabilize the endosomal membrane integrity, finally leading to pore formation and content release [161,162]. By increasing the efficiency of endosomal escape, lower doses of drugs and a reduced number of administrations could be employed, decreasing immunogenicity and side effects, while retaining therapeutic effectiveness.

Cell penetrating peptides (CPPs) are 10–16 amino acids peptides originating from parts of viral, bacterial, insect and mammalian proteins. They are mainly structurally different from each other with no consensus sequence [163]. Among the most widely studied is the protein transduction domain TAT from the human HIV1 transcriptional activator protein Tat, *Drosophila* penetratin, herpes simplex virus structural protein VP22, a short amphipathic peptide Pep-1 and the PreS2-domain of hepatitis B virus surface antigen and a membrane translocation sequence from the human Kaposi. Three different CPPs were fused to dianthin and two of them increased the cytotoxicity of a toxin-based conjugate [164]. A fusion protein between the PreS2-domain and saporin exhibited an enhanced anti-tumor effect in a breast cancer mouse model [165]. Another CPP when appended to Gelonin, the resulting conjugate exhibited significantly improved tumoricidal effects and was shown to inhibit tumor growth in a xenograft mouse model of adenocarcinoma [166]. On the other hand, we found that arginine residues in CPP amino acid sequence significantly affect tumor accumulation in in vivo imaging [167], and further optimized methodology for CPP-based RIP delivery to tumor should be established.

In addition to CPPs, other peptides that support drug activation, release and accumulation can be combined with protein toxins for tumor-specific activation. Proteinase recognition sequence [168], endosomal translocation motif derived from diphtheria toxin [169], and cellular protein retention signals such as KDEL have been used to improve ricin A chain and PEA cytotoxicity [170].

Another way investigated to disrupt endosomal membranes favoring the drug escape is represented by the viral-derived fusogenic peptide GALA, a 30 amino acid peptide formed by a glutamic acid-alanine-leucine-alanine repeat. When pH drops to 5 in the acidified endosome, a conformational change occurs in GALA peptide leading to the formation of an amphipathic alpha-helix that binds to the membrane, forms a transmembrane pore and causes bilayer disassembling [171]. Moreover, non-peptidic substances able to buffer protons and swell when protonated, such as tertiary amine groups, cationic polymers, polyamidoamine dendrimers can be utilized to obtain endosome leakage through the so-called “proton sponge effect” [172]. To maintain the acidic pH, endosomes replace protons captured by cargo by generating an influx of protons and therefore of chloride ions and water, resulting in an osmotic rupture of the membrane [173]. Our research group developed a novel L17E endosomolytic peptides derived from the cationic and membrane-lytic spider venom peptide M-lycotoxin, showing that a combinatorial treatment of the peptide with saporin significantly enhanced saporin biological activity [174].

Saponins, from Latin soap, are plant-derived compounds that can be mainly divided into two groups depending on their chemical structure, steroids and triterpenoids, the latter bringing linear, as well as, branched sugar chains. Most saponins show hemolytic and membrane permeabilizing activity, which are employed by plants as defensive strategy from predators. Glycosylated triterpenoid saponins were demonstrated to substantially increase the effect of plant RIPs saporin and dianthin in murine mammary adenocarcinoma and colon carcinoma, respectively, by improving the transfer of the toxic compound into the cytosol upon co-administration [175,176,177]. Side effects like loss of body weight, altered blood parameters and immune response were only moderate and commonly reversible [178]. However, a main problem with the therapeutic use of saponins, as well as for other endosomal escape enhancers, is that they behave independently compared to the targeted toxins when they are co-administrated in terms of absorption, biodistribution, metabolism and excretion, aside from cellular internalization and release [179]. Hence, the major challenge is to synchronize the enhancer and the toxin effect to warrant that both are present simultaneously at the site of interaction. Several studies investigated the timing of co-administration of saponins and targeted toxins to obtain the utmost combined effect, avoiding off-target effects.

Another way to promote escape from endo-lysosomal compartments consists of light activation of an amphiphilic sensitizer taken up by the target cells, known as photochemical internalization (PCI) [180]. PCI was established as a drug delivery technology for the release of molecules that are sequestered in endosomes after endocytosis leading to the formation of reactive oxygen species (ROS), thus causing lipid peroxidation, protein oxidation and finally resulting in the rupture of endo-lysosomal vesicles and thus cytosolic release of the content [181]. This PCI technique has been successfully applied in vivo. For enhanced endosomal escape of the CD133-targeting immunotoxin AC133 mAb-saporin conjugate (PCI_AC133–saporin_), Bostad et al. developed and showed that AC133-saporin co-localizes with the PCI-photosensitizer TPCS_2a_, which upon light exposure induces cytosolic release of AC133-saporin. In vivo studies, targeting of CD133+ WiDr tumors with PCI_AC133–saporin_ after one cycle of systemic injection and light exposure showed significant delay in tumor growth [182]. An experimental study is under way in Rosemblum’s lab to test the enhancement effects of PCI on VEGF121-rGelonin fusion [183], since, contrary to the triterpenoid saponins, which are selective in their interaction for few, type I RIPs, this technique may apply to promoting endosomal efflux of any endocytosed molecule. PCI of EGF-Gelonin resulted in inhibitory effects on squamous cell carcinoma in a mouse xenograft model and reduction of tumor perfusion and necrosis induction in head and neck squamous cell carcinoma tumors [184].

Recently, a mutant saporin, where a cysteine has been introduced instead of an Ala 157 in an external loop, has been generated to allow conjugation with a small inhibitor molecule targeting integrins αvβ3/αvβ5. This conjugate was demonstrated to be highly cytotoxic and selective in breast cancer and melanoma cell lines. In addition, a tricomponent conjugate in which another cytotoxic agent, auristatin F, a potent microtubule inhibitor, was inserted between saporin and the small integrin inhibitor was tested showing it still maintained high activity and preserved specificity, but was not more effective in vitro as the first conjugate tested, deserving further investigation in xenograft tumor models [185]. This small-molecule RIP bioconjugate approach is highly versatile since it could be broadly applied using other small molecules towards different cancer-associated targets that are already available in large numbers [186].

### 7.6. Employment of Plant Type I RIPs in Other Pathological Models

Instead of anti-cancer treatment, RIPs have been also used to eliminate the cholinergic inputs to hippocampus [187], monocyte-derived inflammatory dendritic cells [188], and alloreactive CD4^+^ and CD8^+^ T-cells through CD137-mediated internalization [189]. The cholinergic projections to ventral subiculum were selectively eliminated using 192 IgG-saporin conjugates, and this model is useful for investigation of the cholinergic modulation of subicular theta-gamma activity on spatial learning and memory functions in rats. Eliminations of cholinergic inputs to ventral subiculum significantly reduced the subicular theta and enhanced the gamma activity during active wake and REM sleep states. The spatial learning was also severely impaired following cholinergic elimination of ventral subiculum [190]. Monocytes play crucial roles for the regulation of tissue homeostasis and the control of pathogens. However, accumulation of monocyte-derived inflammatory dendritic cells participates in the pathogenesis and persistence of inflammatory diseases including psoriasis and Crohn’s disease. Alonso et al. developed a strategy for avoidance of the monocyte intermediates to deplete inflammatory dendritic cells through anti-CD209 antibody conjugated to saporin. Mice with an abundance of inflammatory dendritic cells as a consequence of lipopolysaccharide exposure were treated with anti-CD209 antibody conjugated to saporin, resulting in depletion of CD209 positive dendritic cells [188]. Graft-versus-host disease (GVHD) shows a major limitation to allogeneic hematopoietic stem cell transplantation. CD137 signaling is considered to be involved in multiple stages of GVHD development. Lee et al. developed a method to selectively eliminate alloreactive CD4^+^ and CD8^+^ T-cells through CD137-mediated internalization of anti-CD137 mAbs conjugated with saporin [189]. CD137-expressing cells were killed by the treatment with the conjugate, and transfer of donor T-cells after allodepletion showed no evident GVHD [189].

Substance P-saporin (SP-SAP) deserves a special mention. This is a neuropeptide-toxin conjugate aimed at selectively destroying neurons expressing the pain-related receptor for substance P (neurokinin-1 receptor, NK-1R). It is currently in Phase I clinical study recruiting terminal cancer patients with intractable pain (NCT02036281). Even if nociception is a complex process and substance P is not the only factor involved, intrathecal administration of SP-SAP has been shown to cause a robust change in a variety of pain states in animal models, including dogs [191,192]. In addition, the lumbar delivery of SP-SAP, but not SAP alone, in dogs resulted in a specific, dose-dependent reduction of superficial NK-1R bearing lumbar neurons. Notably, adverse effects in animal behavior, chronic motor functions disturbance, changes in blood pressure or heart rate, progression of the injury or any evidence of additional loss of neurons or function were not detected. Pharmacokinetic studies demonstrated that SP-SAP is cleared relatively rapidly from the cerebrospinal fluid with a clearance of over 50% occurring within 30–60 min [193].

## 8. Conclusions and Perspectives

A few hundred RIP-based ITs and chimeric fusions have been developed so far, but only a bacterial-derived toxin, Denileukin diftitox, has been approved by the FDA as a cancer therapy agent. Most of the clinical studies did not progress beyond Phase 1 study, due to the triggering of immune responses and considerable side effects, such as VLS. Hematologic malignancy patients have experienced on average somewhat less of these problems being immunocompromised; however, after a cycle of IT administration, half to almost 100% of patients treated having solid tumors develop anti-toxin antibodies. Efforts focused to reduce such negative effects are aimed to increase tumor penetration capability in solid tumors, while maintaining the target selectivity, by employing humanized single chain antibody fragments, peptides or small molecules and by masking antigens through PEGylation approaches. Biotechnology approaches as to limit VLS and RIP immune responses with de-immunization strategies, as with the Bouganvilleae de-bouganin toxin, should be pursued and prioritized. Nanotechnology is contributing in this direction by providing nanovectors able to increase the half-life of drugs, to bypass the hydrophobic nature of some molecules, to deliver multiple therapeutics and/or diagnostic agents and to reduce administration by controlled or sustained release of the drug. Along these lines, exosomes are emerging as suitable carriers of biological materials including toxins, providing a safe delivery route. The target selectivity can be achieved by modifying the vesicles, but also by engineering producing cells, increasing the number of possible combinations for the preparation of therapeutic agents. RIPs keep their potential unaltered with these new technologies, altogether opening further to clinical development opportunities.
